# Impact of dental caries and Self-perceived oral health on daily lives of children and mothers in rural Egypt: a household survey

**DOI:** 10.1186/s12903-024-04454-9

**Published:** 2024-08-02

**Authors:** Nourhan M. Aly, Merna Ihab, Nour Ammar, Maryam Quritum, Hana Moussa, Maha El Tantawi

**Affiliations:** 1 Department of Pediatric Dentistry and Dental Public Health, Faculty of Dentistry, Alexandria University, Champollion St, Azarita, Alexandria 21527 Egypt; 2 Department of Conservative Dentistry and Periodontology, University Hospital, Maximilian University of Munich, Ludwig, Germany

**Keywords:** Daily life, Smiling, Social participation, Rural population, Dental caries

## Abstract

**Background:**

The present study assessed the impact of oral health on the daily lives of children and mothers living in a rural area in Northwestern Egypt.

**Methods:**

A cross-sectional household survey including children between 6 and 12 years old and their mothers was conducted in rural Egypt, 2019–2020. Data were collected using clinical examination and interview-based questionnaires of children and mothers. Three binary logistic regression models were used to assess the relationship between the dependent variables (oral health impact (yes, no) on avoiding smiling, chewing problems, and missing school (children) and avoiding social events (mothers)), and the explanatory variables: oral health (clinically-assessed caries experience and self-reported oral health) controlling for sociodemographic profile (child age and sex, mother’s education), daily toothbrushing and village of residence.

**Results:**

A total of 211 households with 355 children and 211 mothers were included (91.5% response rate). About 54% of the children were girls, mean (SD) age = 8.7 (2.05) years and 82.3% did not brush their teeth daily. Mother’s mean (SD) age was 31.70 (5.45) years. Because of dental problems, 31.3% of children reported chewing difficulties, 31% avoided smiling compared to 76.3% and 43.6% of mothers. Also, 30.4% of children missed school and 76.8% of mothers reported reduced participation in social activities. In children, the number of decayed anterior teeth was associated with significantly higher odds of avoiding smiling (AOR = 1.22, 95%CI: 1.03, 1.44). In mothers, a greater number of posterior missing teeth was associated with significantly higher odds of chewing difficulties (AOR = 1.21, 95%CI: 1.01, 1.45), and a greater number of all missing teeth was associated with significantly higher odds of reduced participation in social events (AOR = 1.30, 95%CI: 1.30, 1.57). Good/ very good reported oral health in children and mothers was associated with lower odds of avoiding smiling and chewing problems (*p* < 0.05).

**Conclusion:**

Decayed anterior teeth in children have a negative impact on smiling whereas missing teeth in mothers affect the ability to chew food and socialize. The psychological, functional, and social impacts of caries in this rural setting needs to be mitigated by improving oral health literacy and access to care.

**Supplementary Information:**

The online version contains supplementary material available at 10.1186/s12903-024-04454-9.

## Introduction

Oral health is a fundamental part of overall health and a determining factor of an individual’s quality of life. The connection between oral and overall health extends beyond the oral cavity [1]. In young age, childhood is a pivotal period during which the foundations for physical, emotional, and social well-being are established and are influenced by a child’s oral health with effects extending well into adulthood [2, 3]. This is particularly relevant to low and low-middle income countries, where caries is highly prevalent [4]. The latest national oral health survey supported by the World Health Organization (WHO) in Egypt showed that over 70% of Egyptian children and adults suffered from untreated caries [5, 6].

Compromised oral health can lead to many problems, with extensive consequences that negatively impact various aspects of an individual’s life [7]. If left untreated, dental caries can cause persistent pain that disrupts daily life. This pain can hinder children’s ability to concentrate on their studies and impede their educational progress, by leading them to miss days of school [8]. Similarly, adults may experience reduced productivity due to dental pain [9]. If dental decay remains unaddressed, oral health problems increase, including infection and tooth loss with irreversible consequences [9, 10].

Advanced dental decay may also lead to a decline in masticatory efficiency and speech articulation through several mechanisms [11, 12]. Individuals suffering from dental pain may use soft-textured or processed foods and avoid chewy foods that have good nutritional value to avoid pain. This may contribute to malnutrition and obesity along with general health problems [13, 14]. Additionally, mispronunciation and speech difficulties resulting from dental caries can hinder a child’s development and potentially subject them to bullying [15].

Notably, the relationship between compromised oral health and social interaction is a multifaceted issue with profound implications for individuals’ quality of life and overall well-being. A recent systematic review showed that adults with poor oral health had an odds ratio of 1.47 for social isolation and looniness in comparison to those with better oral health [16]. Similarly, a population-based study concluded that a higher prevalence of tooth loss in patients was associated to a negative impact on their engagement in social activities [17]. Furthermore, dental caries may affect self-confidence, the capacity to foster interpersonal relationships, and mental well-being [18, 19]. This is particularly relevant in cases of visible caries and loss of tooth structure in the anterior region, especially in young children [20, 21]. This may deter children from smiling and engaging in normal interactions with peers, which are vital components of a child’s social development [22]. As for adults, the appearance of dental decay can foster feelings of self-consciousness and insecurity which may promote dissatisfaction [23]. These social impacts amplify the social repercussions of dental decay, with children missing school days and adults avoiding participation in social events [17].

Most Egyptians live in rural areas characterized by high poverty rates and limited access to education and healthcare [24]. This places this population at high risk of oral diseases with negative impact on their daily lives. It may be argued, however, that the daily challenges facing this disadvantaged population may overshadow the impact of oral health on lives. Context-specific evidence on this impact is important to plan dental care services and dental health education programs. Notably, few studies have investigated this research area beyond Western and Southeast Asian nations [25, 26], highlighting the need for region-focused perspectives. Thus, the present study assessed the impact of clinically assessed and self-reported oral health on the daily lives of children and mothers living in rural Egypt. The null hypothesis was that there would be no impact of oral health on the daily lives of mothers and children in this population.

## Methods

### Study design

This was a secondary analysis using data from a household survey assessing the association between parenting practices and the oral health status of children residing in rural areas in Egypt [27]. The original study was a cross sectional household survey from May 2019 to February 2020. The study received ethical approval from the Ethics Committee of the Faculty of Dentistry, Alexandria, Egypt (IRB 00010556 – IORG 0008839). Parental informed consent and children’s assent were prerequisites for participation. The study was carried out according to the principles of the Declaration of Helsinki [28] and followed the STrengthening the Reporting of OBservational studies in Epidemiology (STROBE) reporting guidelines [29].

### Study setting and participants

**Fig. 1:**
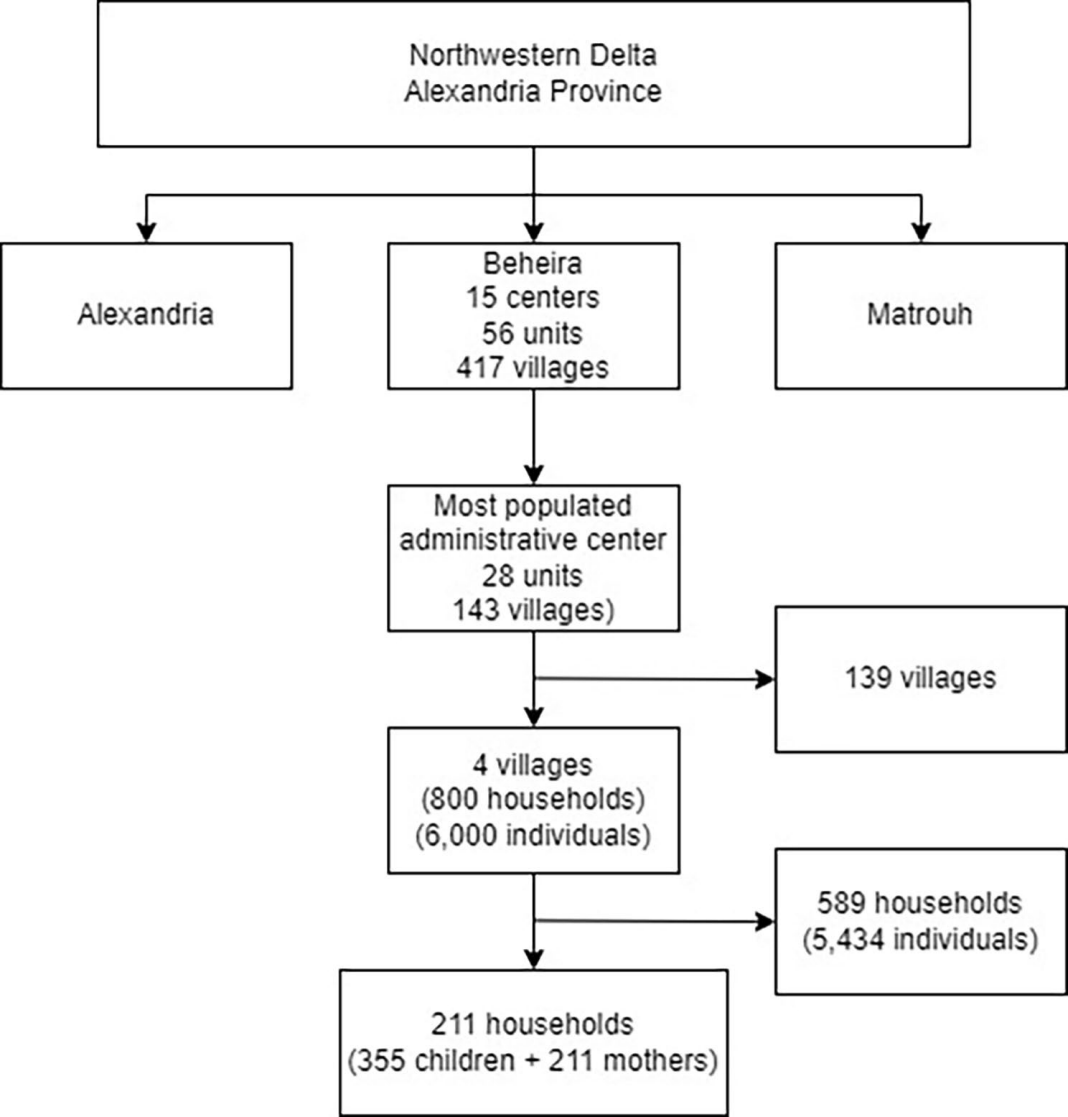
Overview of the study multistage sampling strategy

The study was conducted in the Northwestern Delta region of Egypt. Multistage cluster sampling was used. Initially, the most populous administrative center in the region was randomly chosen. The center has 28 administrative units, and these units include villages. In the second stage, villages within the administrative units were identified and four of them were randomly selected. In the third stage, a local guide assisted in the random selection of eligible households and a cluster sample was used to include all eligible children and their mothers in the selected households (Fig. 1).

The original study determined the sample size based on 95% confidence level and 80% study power to detect caries among Egyptian children, with a mean (SD) DMFT of 1.04 (1.56) and mean (SD) dft of 4.21 (3.21) [30]. The required sample was 370 children. We made sure that the present study was adequately powered to detect at least a small effect size based on an odds ratio = 1.5 [31] or 1.68 [32] of the impact of oral health on daily lives assuming different distributions of the independent variables where a maximum of 179 participants would be needed.

The criteria for including children in the study were: (1) aged 6–12 years old, (2) living in villages in rural areas in Northwestern Egypt, (3) free from medical or intellectual disabilities, (4) with mother or female caregiver available for participation, and (5) mothers/ female caregivers in the same household as the child. Consenting mothers of eligible children were included. Pre-school children (< 6 years old), those with medical and intellectual disabilities, and who did not live with their mother/ female caregiver in the same household were excluded.

### Data collection

Data were collected from mothers and children using clinical examination and interview-based questionnaires. Clinical examination for caries followed the World Health Organization (WHO) criteria [33] under natural daylight using a ball ended WHO probe #550B and a disposable plane mouth mirror. The numbers of primary and permanent teeth with caries experience were recorded for the children and mothers. Two calibrated dentists conducted the clinical examination with Kappa statistic for intra- and inter-examiner reliability ranged from 0.85 to 0.94.

Interview-based questionnaires using the Arabic version of WHO child and adult questionnaires [33] were used for data collection **(Appendix 1).** The first section collected data on background information (child sex and age in years, highest educational level of the mother and mother’s age), oral health practices including toothbrushing frequency (at least once daily or less), self-reported teeth health (excellent, very good/good, average, and poor). The second section of the questionnaire was designed based on the Arabic version of WHO child and adult questionnaires which have been previously validated [33]. The questions were adapted to assess assessed the impact of oral health on daily living of children and mothers. Children were asked to respond with ‘yes’ or ‘no’ to questions regarding whether they avoided smiling due to dental problems, missed school or classes because of teeth problems, and experienced difficulties with chewing. Mothers were also asked to respond using ‘yes’ or ‘no’ if they avoided smiling because of teeth problems, experienced chewing difficulties, and if their participation in social events decreased due to dental problems.

To ensure validation, the questionnaire underwent a rigorous validation process. This process involved pilot testing, expert review, and statistical analysis to assess construct validity, and content validity. Additionally, we considered cross-cultural adaptability by modifying language and format to ensure comprehension and accuracy of responses.

The questionnaires were uploaded on the online platform Kobo toolbox [34]. The survey was then pre-tested on ten families, mothers, and children, attending the pediatric dentistry clinic of the Faculty of Dentistry, Alexandria University to ensure that the questions were clearly articulated and that the response options were relevant and comprehensive. The survey was further pilot tested amongst seven households, mothers, and children, in a village before being administered on a larger scale.

Following clinical examinations and questionnaire administration, other family members not included in the study were screened for oral diseases and referred for treatment as needed. Each family was also given incentives in the form of toothbrushes and toothpastes.

### Statistical analysis

**Fig. 2:**
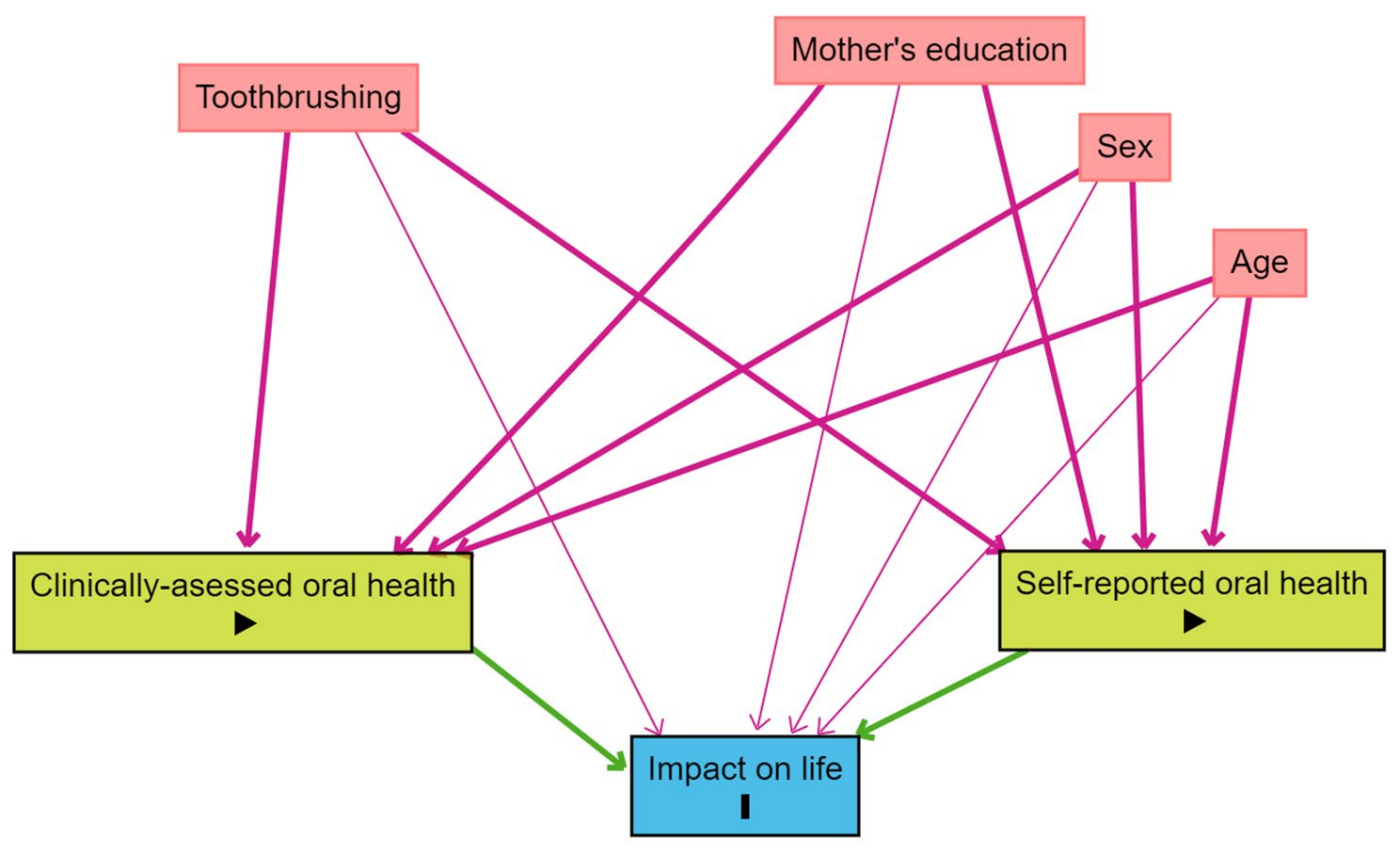
Conceptual model of the study variables (DAG)

Data were analyzed using IBM SPSS for Windows (Version 26.0, IBM Corp.) and significance was inferred at *p* value < 0.05. The dependent variables were the oral health impact on daily lives including avoiding smiling, difficulty chewing and, for children, missing school and for mothers, reduced participation in social activities. The independent variables were oral health indicators, clinically assessed caries experience and self-reported oral health. The confounders were daily toothbrushing, and socioeconomic factors (child and mother age, child sex, and mother education), and village of residence. The relation between the dependent and independent variables is shown in the directed acyclic graph (DAG) in Fig. 2. Three binary logistic regression models were used, one for each impact on daily life, for the mothers and for the children to assess the association between the dependent and independent variables after adjusting for the confounders and village of residence. We assessed multi-collinearity and identified strong correlations (*r* > 0.7) among several variables. Consequently, variables consistent with the study’s conceptual framework were incorporated in the regression models. Adjusted odd ratios (AORs), 95% confidence intervals (CIs), and Nagelkerke’s R^2^ were calculated.

## Results

**Fig. 3:**
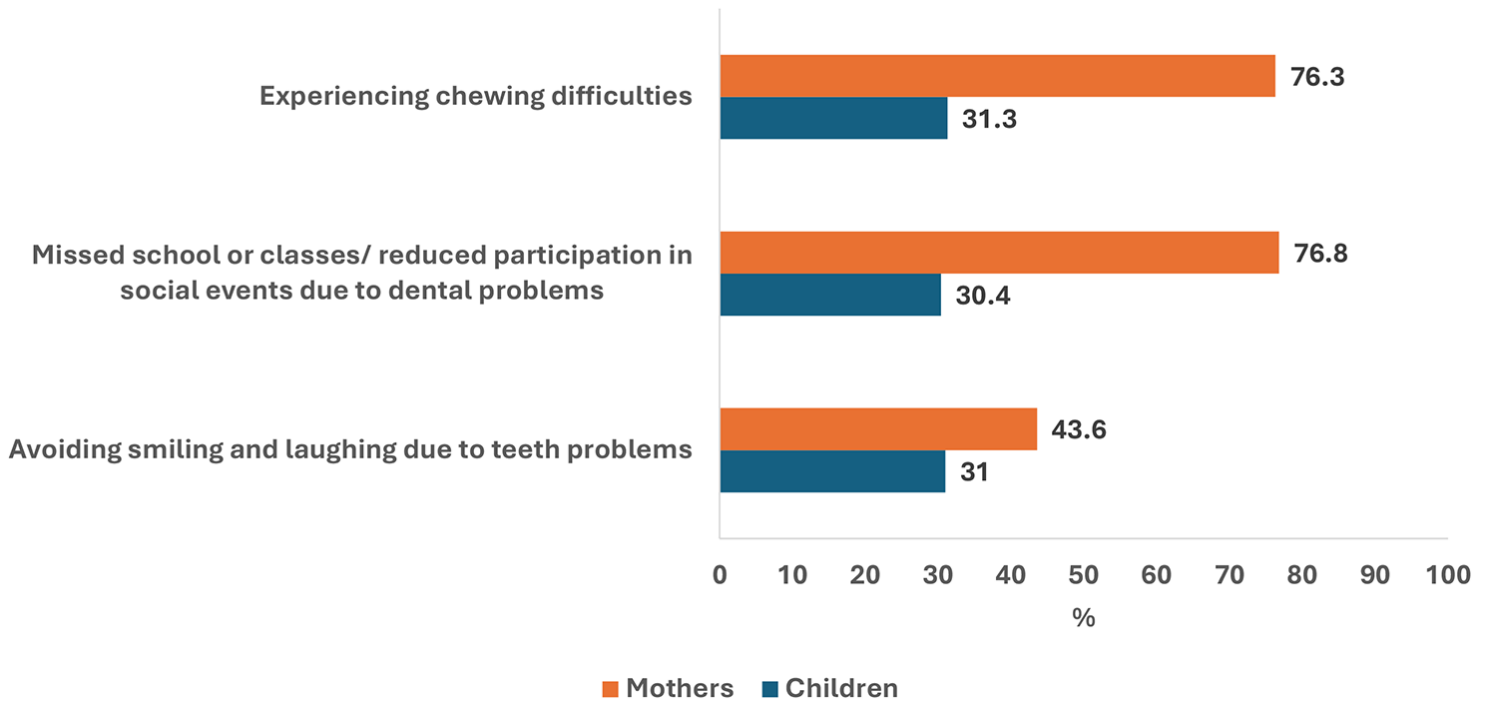
Impact of oral health on daily living in children and mothers

In the four villages, a total of 211 households with 355 children and 211 mothers were included in this study (response rate = 91.5%). Table 1 shows the main characteristics of the children and their mothers. Girls represented 54% of the children, children’s mean (SD) age = 8.7 (2.05) years and 17.7% brushed their teeth once daily. The mother’s mean (SD) age was 31.70 (5.45) years and 14.7% completed high school education or more. Also, 38.9% and 19% of the children and mothers reported very good / good teeth health, respectively. The children’s mean number of teeth with caries experience (SD) was 3.79 (3.19), and the mean number of teeth with caries experience in mothers mean (SD) was 6.88 (5.35). Because of dental problems, 31% of children avoided smiling, 30.4% missed school and 31.3% reported chewing difficulties. Among mothers, 43.6% avoided smiling, 76.8% reduced participation in social activities and 76.3% experienced chewing difficulties (Fig. 3**).**

**Table 1:** Characteristics of children and mothers included in the study

Children (*n* = 355)	Age (years)	Mean (SD)	8.7 (2.1)
	Sex: n (%)	Boys	164 (46.2)
		Girls	191 (53.8)
	Daily toothbrushing: n (%)	Yes	63 (17.7)
		No	292 (82.3)
	Self-reported teeth health: n (%)	Excellent	0 (0%)
		Very good / good	138 (38.9)
		Average	110 (31.0)
		Poor	107 (30.1)
	Number of teeth with caries experience	decayed anterior teeth: mean (SD)	0.70 (1.52)
		decayed posterior teeth: mean (SD)	2.97 (2.25)
		All decayed teeth: mean (SD)	3.68 (3.16)
		filled anterior teeth: mean (SD)	0.01 (0.13)
		filled posterior teeth: mean (SD)	0.09 (0.43)
		All filled total: mean (SD)	0.10 (0.44)
		dft index: mean (SD)	3.79 (3.19)
Mothers (*n* = 211)	Age (years)	Mean (SD)	31.7 (5.5)
	Mother education: n (%)	Non-educated	78 (37.0)
		Completed primary or middle school	102 (48.3)
		Completed high school or more	31 (14.7)
	Self-reported teeth health: n (%)	Excellent	0 (0%)
		Very good / good	40 (19.0)
		Average	52 (24.6)
		Poor	119 (56.4)
	Number of teeth with caries experience	Decayed anterior teeth: mean (SD)	0.43 (1.02)
		Decayed posterior teeth: mean (SD)	3.00 (2.46)
		All Decayed teeth: mean (SD)	3.43 (2.79)
		Missing anterior teeth: mean (SD)	0.05 (0.36)
		Missing posterior teeth: mean (SD)	2.74 (0.07)
		All Missing teeth: mean (SD)	2.79 (3.19)
		Filled anterior teeth: mean (SD)	0.14 (0.61)
		Filled posterior teeth: mean (SD)	0.77 (1.66)
		All Filled teeth: mean (SD)	0.91 (1.92)
		DMFT index: mean (SD)	6.88 (5.35)

Table 2 represents the impact of children’s oral health on avoiding smiling, chewing problems, and missing school controlling for confounders. The number of decayed anterior teeth was significantly associated with higher odds of avoiding smiling (AOR = 1.22, 95% CI: 1.03, 1.44). Compared to reporting poor teeth health, reporting very good/ good teeth health was associated with significantly lower odds of avoiding smiling (AOR = 0.34, 95% CI: 0.18, 0.64), chewing problems (AOR = 0.32, 95% CI: 0.17, 0.61) and missing school (AOR = 0.54, 95% CI: 0.30, 0.997). All three models correctly classified > 70% of children and the highest variation was explained by variables in the avoiding smiling model (Nagelkerke’s R^2^ = 0.146).

**Table 2:** Association between oral health status and impact on daily living in children

		Avoided smiling		Difficulty chewing		Missed school	
		AOR (95% CI)	*P* value	AOR (95% CI)	*P* value	AOR (95% CI)	*P* value
Child age		1.17 (1.02, 1.35)	0.02	1.12 (0.97, 1.30)	0.11	1.01 (0.88, 1.17)	0.88
Child sex: Boys vs. girls		0.68 (0.41, 1.14)	0.14	0.72 (0.43, 1.20)	0.20	1.09 (0.66, 1.80)	0.74
Mother age		1.02 (0.97, 1.07)	0.55	1.01 (0.96, 1.06)	0.67	1.01 (0.96, 1.06)	0.84
Mother education	Non-educated vs. high school or more	1.06 (0.48, 2.33)	0.88	1.06 (0.49, 2.30)	0.89	0.47 (0.23, 0.96)	0.04
	Primary or middle vs. high school or more	1.09 (0.50, 2.34)	0.83	1.06 (0.49, 2.26)	0.89	0.41 (0.20, 0.82)	0.01
Daily toothbrushing: Yes vs. no		1.07 (0.55, 2.08)	0.84	1.08 (0.55, 2.10)	0.83	1.81 (0.97, 3.37)	0.06
Self-reported teeth health	Very good/ good vs. poor	0.34 (0.18, 0.64)	0.001	0.32 (0.17, 0.61)	< 0.001	0.54 (0.30, 0.997)	0.049
	Average vs. poor	0.89 (0.49, 1.64)	0.72	0.88 (0.48, 0.61)	0.68	0.89 (0.48, 1.64)	0.71
Number of decayed anterior teeth		1.22 (1.03, 1.44)	0.02	-		-	
Number of decayed posterior teeth		-		1.03 (0.90, 1.17)	0.68	-	
Number of all decayed teeth		-		-		1.03 (0.95, 1.13)	0.47
Number of all filled teeth		-		-		0.97 (0.53, 1.75)	0.91
Model X^2^ (*p* value)		36.30 (< 0.001)		31.01 (0.002)		15.60 (0.27)	
Nagelkerke’s R^2^		0.146		0.126		0.065	
Percent correctly classified		71.6%		71.9%		71.3%	

AOR: Adjusted Odds Ratio, CI: Confidence interval. Models adjusted for village of residence

Table 3 shows the impact of oral health on mothers’ daily living after adjusting for confounders. Mothers who reported their teeth to be in good or very good health had significantly lower odds of avoiding smiling (AOR = 0.37, 95% CI: 0.16, 0.87), chewing difficulties (AOR = 0.31, 95% CI: 0.12, 0.81) but not reduced social activities (AOR = 0.95, 95% CI: 0.35, 2.58) than those reporting poor teeth health. The number of missing posterior teeth was associated with significantly higher odds of chewing difficulties in mothers (AOR = 1.21, 95% CI: 1.01, 1.45). Also, the number of all missing teeth was associated with significantly higher odds of decreased participation in social events (AOR = 1.30, 95% CI: 1.07, 1.57). The reduced social activity model had the highest percentage of correctly classified (81.5%) and the chewing difficulties model had the highest portion of explained variance (Nagelkerke’s R^2^ = 0.269).

**Table 3:** Association between oral health status and impact on daily living in mothers

		Avoided smiling		Chewing problems		Reduced social activities	
		AOR (95% CI)	*P* value	AOR (95% CI)	*P* value	AOR (95% CI)	*P* value
Age		0.94 (0.89, 1.00)	0.07	0.40 (0.11, 1.47)	0.17	0.96 (0.90, 1.04)	0.32
Mother education	Non-educated vs. high school or more	0.42 (0.17, 1.06)	0.07	0.59 (0.17, 2.13)	0.43	1.33 (0.43, 4.09)	0.62
	Primary or middle vs. high school or more	0.55 (0.22, 1.35)	0.19	0.98 (0.92, 1.06)	0.67	0.93 (0.32, 2.73)	0.89
Daily toothbrushing: Yes vs. no		0.66 (0.29, 1.55)	0.34	0.40 (0.16, 0.97)	0.04	0.57 (0.23, 1.40)	0.22
Self-reported teeth health	Very good/ good vs. poor	0.37 (0.16, 0.87)	0.02	0.31 (0.12, 0.81)	0.02	0.95 (0.35, 2.58)	0.93
	Average vs. poor	0.43 (0.20, 0.92)	0.03	0.21 (0.08, 0.51)	0.001	0.48 (0.21, 1.11)	0.09
Number of decayed anterior teeth		1.08 (0.80, 1.46)	0.61	-		-	
Number of decayed posterior teeth		-		1.01 (0.85, 1.19)	0.94	-	
Number of all decayed teeth		-		-		1.08 (0.92, 1.26)	0.35
Number of missing anterior teeth		3.90 (0.39, 39.16)	0.25	-		-	
Number of missing posterior teeth		-		1.21 (1.01, 1.45)	0.04	-	
Number of all missing teeth		-		-		1.30 (1.07, 1.57)	0.007
Number of all filled teeth		-		-		-	
Model X^2^ (*p* value)		31.46 (0.001)		41.55 (< 0.001)		34.00 (< 0.001)	
Nagelkerke’s R^2^		0.186		0.269		0.225	
Percent correctly classified		69.2%		76.8%		81.5%	

AOR: Adjusted Odds Ratio, CI: Confidence interval. Models adjusted for village of residence

## Discussion

The current study showed that in a rural setting in Egypt, oral health affected the daily lives of children and mothers, with a higher proportion of mothers than children affected and more effect on chewing and participation in social activities than on smiling among mothers. In children, more decayed anterior teeth were associated with higher odds of avoiding smiling. More missing posterior teeth were associated with higher odds of mothers having a chewing problem and a greater number of all missing teeth was associated with higher odds of reduced participation in social activities. Good or very good self-reported oral health was associated with lower odds of avoiding smiling and chewing problems in children and mothers and lower odds of missing school among children but not lower odds of participation in social activities among mothers. Overall, oral health had considerable impact on the daily lives of children and mothers, and thus, the null hypothesis can be partially rejected.

The study had some limitations. The cross-sectional design of the study cannot confirm causation but can only suggest association. Also, we could not interview children younger than 6 years of age because of their limited attention span and underdeveloped cognitive abilities. Another limitation is the lack of data collection regarding some potential confounders such as trauma to the anterior teeth, malalignment of the anterior teeth, malocclusion, and other related factors. This limitation may have influenced the interpretation of our results, as these variables could have had an impact on the outcomes under investigation. In addition, there may be a potential risk of recall and social desirability biases associated with the use of questionnaires. Finally, the study was conducted in one location of rural Egypt, and caution should be applied when applying the findings to other regions or populations. Nevertheless, the study has several strengths including using a household survey that offers a sample that closely represents the larger population, enhancing the generalizability of findings. By using a validated questionnaire with demonstrated cross-cultural adaptability, we bolstered the robustness of our study’s findings, enhancing the credibility and generalizability of our results to the target population. Also, the use of interview-based questionnaires allowed for the inclusion of non-educated mothers, resulting in a higher response rate and more generalizable findings. The study contributes to the existing body of knowledge by addressing a gap in oral health research related to the impact of oral health problems on rural populations. Research often focuses on urban or more developed regions, which may not accurately represent the unique challenges faced by rural populations. Thus, this study offers insights that can help plan oral health interventions and policies for underserved communities in Egypt and potentially other rural areas worldwide when applicable.

The study has some important findings. First, children with more decayed anterior teeth had significantly higher odds of avoiding smiling, and about one out of three children avoided smiling. The finding indicates that children may be self-conscious of their appearance, including their teeth [25, 35] resulting in a reluctance to smile due to societal pressure disapproving of bad teeth [36]. These findings agree with previous studies [20, 36, 37] reporting that dental caries can have a serious impact on smiling especially when the decay is in the anterior teeth. The potential effect of this problem on self-image, self-confidence, interaction with peers and bullying may suggest an association between oral and mental health at this young age that warrants further investigation.

Second, mothers with more missing posterior teeth had higher odds of reporting chewing problems. At the end of their third decade, the mothers participating in the study had already lost, on average, three teeth because of caries with three more decayed teeth expected to be lost afterwards considering the low level of dental care observed among participants. Posterior teeth are important for chewing, and their absence can impact the ability to effectively process and breakdown food [38]. This impaired chewing ability may promote the use of energy-dense, nutrient-poor foods [13], especially if these foods are available at lower prices [39] with tooth loss leading to malnutrition especially obesity [13]. The intersection between high caries severity and chewing problems in Egyptian women may have implications for the sharply increasing problem of overweight in Egyptian women [40] and warrants further investigation.

Third, a greater number of missing teeth was associated with significantly decreased participation of mothers in social events. Tooth loss can lead to feelings of embarrassment and insecurity, affecting one’s willingness to participate in social activities leading to social withdrawal and isolation. Beside esthetics, tooth loss can also affect an individual’s ability to eat and speak, which can be noticeable during meals or conversation in social events, deterring individuals from participating in these events [10, 41]. Less participation in social events may be an indication of social isolation [42]. Studies on older adults show an association between the number of remaining teeth and social isolation [43] with limited evidence on adults [16]. Our findings provide evidence on this association among women of childbearing age, which is especially importance considering the nature of the closely knit rural community where barriers among households are much weaker than in urban communities. However, because of the cross-sectional study design, it is difficult to verify whether socially isolated women who have less participation in social events had more missing teeth due to, for example, their inadequate social network which provided no support to identify dental care opportunities, or women with more missing teeth became more socially isolated because they avoided social events for various reasons related to their dental morbidity. Future longitudinal studies are needed to elucidate this relationship.

Fourth, better self-reported oral health was significantly associated with lower impact on the daily lives of children and mothers, possibly due to less dental pain or problems with improved daily functioning [44]. This agrees with several studies [44–47] indicating that poor self-reported oral health was associated with greater impact on daily living. The only exception was the non-significant association between reduced participation of mothers in social events and self-reported oral health despite the association with the number of missing teeth. This may indicate that social isolation is related to the physical sequels of tooth loss rather than the mother’s perception of their impact. For example, manifest physical or medical problems associated with tooth loss may provide socially acceptable reasons for the mothers to avoid social events, even though they would otherwise be expected to participate as a community duty.

Finally, the more enhanced impact on daily lives of mothers compared to children could be attributed to several factors, including cognitive development and self-awareness. Mothers, being adults, may have a greater understanding of oral health issues and their impact on daily life compared to children. They are also likely to be more conscious of their appearance and social interactions, leading to a higher reported impact on chewing and participation in social activities, which are aspects closely linked to adult responsibilities and social roles [48]. 

These findings collectively shed light on the impact of oral health on the daily lives of children and mothers in a rural setting in Egypt. The high level of untreated decay affected function, social interaction and may be associated with social isolation with possible implications on mental health and other non-communicable diseases associated with food choices. It is important to address these oral health problems using interventions aiming at improving oral health literacy to empower women to control their and their children’s oral diseases and by ensuring better access to dental care services.

### Electronic supplementary material

Below is the link to the electronic supplementary material.

Supplementary Material 1

## Data Availability

The datasets used and/or analyzed during the current study are available from the corresponding author on reasonable request.
