# Supplementary material for: Impact of dental caries and Self-perceived oral health on daily lives of children and mothers in rural Egypt: a household survey
Source: BMC Oral Health. 2024 Aug 2;24:884. doi: 10.1186/s12903-024-04454-9 (PMC11297685; doi:10.1186/s12903-024-04454-9)
Supplement: Supplementary file 1 — Supplementary Material 1 [file 12903_2024_4454_MOESM1_ESM.docx]

**Appendix 1**

**Oral health questionnaire for adults**

1. **Identification number :**
2. **How old are you today (in years) ?**
3. **How would you describe the state of your teeth ?**

- Excellent
- Very good
- Good
- Average

Poor

1. **How often do you clean your teeth?**

- Never
- Once a month
- 2-3 times a month
- 2-6 times a week
- Once a day
- Twice or more a day

1. **Because of the state of your teeth or mouth, how often have you experienced any of the following problems during the past 12 months ?** (Yes, No, I don’t Know)

| - Difficulty chewing foods - Have avoided smiling because of teeth - Have reduced participation in social activities  1. **What level of education have you completed?**  - No formal schooling. - Less than primary school - Primary school completed - Secondary school completed - High school completed - College/university completed - Postgraduate degree |
| --- |

**Oral health questionnaire for children**

1. **Identification number :**
2. **Sex :**

- Boy
- Girl

1. **How old are you today (in years) ?**
2. **How would you describe the health of your teeth?**

- Excellent
- Very good
- Good
- Average
- Poor

1. **How often do you clean your teeth?**

- Never
- Several times a month (2–3 times)
- Once a week
- Several times a week (2–6 times)
- Once a day
- 2 or more times a day

1. **Because of the state of your teeth and mouth, have you experienced any of the following problems during the past year?** (Yes, No, I don’t Know)

- I often avoid smiling and laughing because of my teeth
- Toothache or discomfort caused by my teeth forced me to miss classes at school or miss school for whole days
- I have difficulty in chewing
